# Effective Elicitation of Human Effector CD8^+^ T Cells in HLA-B*51:01 Transgenic Humanized Mice after Infection with HIV-1

**DOI:** 10.1371/journal.pone.0042776

**Published:** 2012-08-06

**Authors:** Yoshinori Sato, Sayaka Nagata, Masafumi Takiguchi

**Affiliations:** Center for AIDS Research, Kumamoto University, 2-2-1 Honjo, Kumamoto, Japan; University Hospital Zurich, Switzerland

## Abstract

Humanized mice are expected to be useful as small animal models for *in vivo* studies on the pathogenesis of infectious diseases. However, it is well known that human CD8^+^ T cells cannot differentiate into effector cells in immunodeficient mice transplanted with only human CD34^+^ hematopoietic stem cells (HSCs), because human T cells are not educated by HLA in the mouse thymus. We here established HLA-B*51:01 transgenic humanized mice by transplanting human CD34^+^ HSCs into HLA-B*51:01 transgenic NOD/SCID/Jak3^−/−^ mice (hNOK/B51Tg mice) and investigated whether human effector CD8^+^ T cells would be elicited in the mice or in those infected with HIV-1 NL4-3. There were no differences in the frequency of late effector memory and effector subsets (CD27^low^CD28^−^CD45RA^+/−^CCR7^−^ and CD27^−^CD28^−^CD45RA^+/−^CCR7^−^, respectively) among human CD8^+^ T cells and in that of human CD8^+^ T cells expressing CX3CR1 and/or CXCR1 between hNOK/B51Tg and hNOK mice. In contrast, the frequency of late effector memory and effector CD8^+^ T cell subsets and of those expressing CX3CR1 and/or CXCR1 was significantly higher in HIV-1-infected hNOK/B51Tg mice than in uninfected ones, whereas there was no difference in that of these subsets between HIV-1-infected and uninfected hNOK mice. These results suggest that hNOK/B51Tg mice had CD8^+^ T cells that were capable of differentiating into effector T cells after viral antigen stimulation and had a greater ability to elicit effector CD8^+^ T cells than hNOK ones.

## Introduction

Humanized mice established by transplanting human CD34^+^ hematopoietic stem cells (HSCs) into immunodeficient mice would be a useful tool for *in vivo* studies of human immune responses, infectious diseases, preclinical testing of vaccines, and new therapeutic strategies. NOD/SCID/IL2rγc^null^ (NOG) [1]–[5], NOD/SCID/IL2rγ^null^ (NSG) [6], [7] and Rag2^−/−^γC^−/−^ mice [8] have been used as good recipients for human cell reconstitution [9], [10], [11]. Studies using such mice have demonstrated the long-term reconstitution and maturation of human T and B cells, as evidenced by the development of Ig-producing human B cells as well as human CD4/CD8 single-positive T cells in the spleen and peripheral blood of the mice. Since the function of human CD8^+^ T cells plays an important role in the elimination of virus-infected cells, the function of these cells in humanized mice has been examined and demonstrated in previous studies [12]. The proliferation and IFN-γ expression of Epstein-Barr virus (EBV)-specific human CD8^+^ T cells have been demonstrated in humanized NOG, NSG, and Rag2^−/−^γC^−/−^ mice established by transplanting human CD34^+^ HSCs into those mice after an EBV infection [8], [13], [14]. However, high-dose injection of EBV causes a fatal lymphoproliferative disorder in humanized NOG mice, whereas a lower-dose injection induces an apparently asymptomatic persistent infection [14], suggesting that the human T cell responses were not able to control the replication of EBV in these mice. Besides, the response of EBV-specific CD8^+^ T cells was identified only occasionally in EBV-infected humanized NSG mice [13]. HCV-specific T cells can be detected in CD34^+^ cell-transplanted NSG mice immunized with a recombinant adenoviral vector encoding the E1 and E2 envelope glycoproteins of HCV, though the response is not observed in all of such mice [15]. On the other hand, an earlier study of ours demonstrated that human CD8^+^ T cells from NOD/SCID/Jak3^−/−^ mice transplanted with human CD34^+^ HSCs (hNOK mice) express perforin and granzyme B at a low level and also fail to produce IFN-γ and to proliferate after stimulation with alloantigens [16], suggesting that these human CD8^+^ T cells can not differentiate into effector cells in such mice. These results indicate that the effector function and antigen-specific responses of human CD8^+^ T cells could not be elicited in immunodeficient mice transplanted with only human CD34^+^ HSCs, because the reconstituted human CD8^+^ T cells were educated by mouse MHC-peptide complexes in the host thymus. Altogether, there has remained an argument about the function of human CD8^+^ T cells in humanized mice.

Several studies showed that antigen-specific responses are detectable in humanized NOD/SCID mice established by transplanting human fetal thymus/liver tissues and CD34^+^ fetal liver cells into them (BLT mice) when the mice are infected with EBV or human immunodeficiency virus (HIV) [17]–[19]. These studies suggest that human T cells require HLA expression on the thymus for developing human immune responses. Indeed, previous studies demonstrated that virus-specific CD8^+^ T cells are elicited in NSG mice expressing HLA-A2 (NSG-HLA-A2/HHD mice): human CD8^+^ T cells from EBV-infected NSG-HLA-A2/HHD mice transplanted with HLA-A2^+^ human CD34^+^ HSCs produce IFN-γ in the presence of target autologous EBV-infected B-lymphoblastoid cell line cells (LCLs) in an HLA-restricted manner [13], [20]. Also, human T cells from dengue virus-infected NSG-HLA-A2/HHD mice transplanted with HLA-A2^+^ human CD34^+^ HSCs are able to secrete IFN-γ, IL-2, and TNF-α in response to stimulation with any of 3 previously identified A2-restricted dengue peptides [21].

There are several studies using humanized mice as small animal models for analysis of HIV-1 pathogenesis [22]. These studies indeed show a high viremia state for more than 3 months and a gradual decrease in the number of human CD4^+^ T cells in HIV-1-infected humanized NOG or Rag2^−/−^γC^−/−^ mice [23]–[26], the production of both anti-HIV-1 Env gp120- and Gag p24-specific antibodies in humanized NOG mice with a high rate of HIV-1 infection [27], the role of Treg cells in the maintenance of high levels of HIV-1 infection in HIV-1-infected humanized Rag2^−/−^γC^−/−^ mice [28], and the selection of mutations by APOBEC3 in the HIV genome in humanized NOG mice [29]. One of these studies demonstrated the presence of HIV gag- and envelope-specific, IFN-γ- and IL-2- producing human CD8^+^ and CD4^+^ cells in the spleens of these mice [26]. In addition, a recent study demonstrated that IFN-γ expression of T cells from 3 out of 5 NSG mice infected with HIV-1 is detectable following stimulation with *gag* or *env* peptides [30]. However, human T cells should be educated by HLA in the thymus of the mice to elicit their suitable function against foreign antigen [16]. Therefore, the function of human CD8^+^ T cells against an HIV-1 infection in the humanized mice has remained controversial.

In the present study, we established HLA-B*51:01 transgenic humanized mice by transplanting human CD34^+^ HSCs into HLA-B*51:01 transgenic NOK (hNOK/B51Tg) mice and focused on analysis of the differentiation of human CD8^+^ T cells in these mice. In addition, we investigated the differentiation of these cells in both hNOK/B51Tg and hNOK mice after an HIV-1 infection.

## Results

### Reconstitution of human T cells in NOK/B51Tg and NOK mice

**Figure 1:**
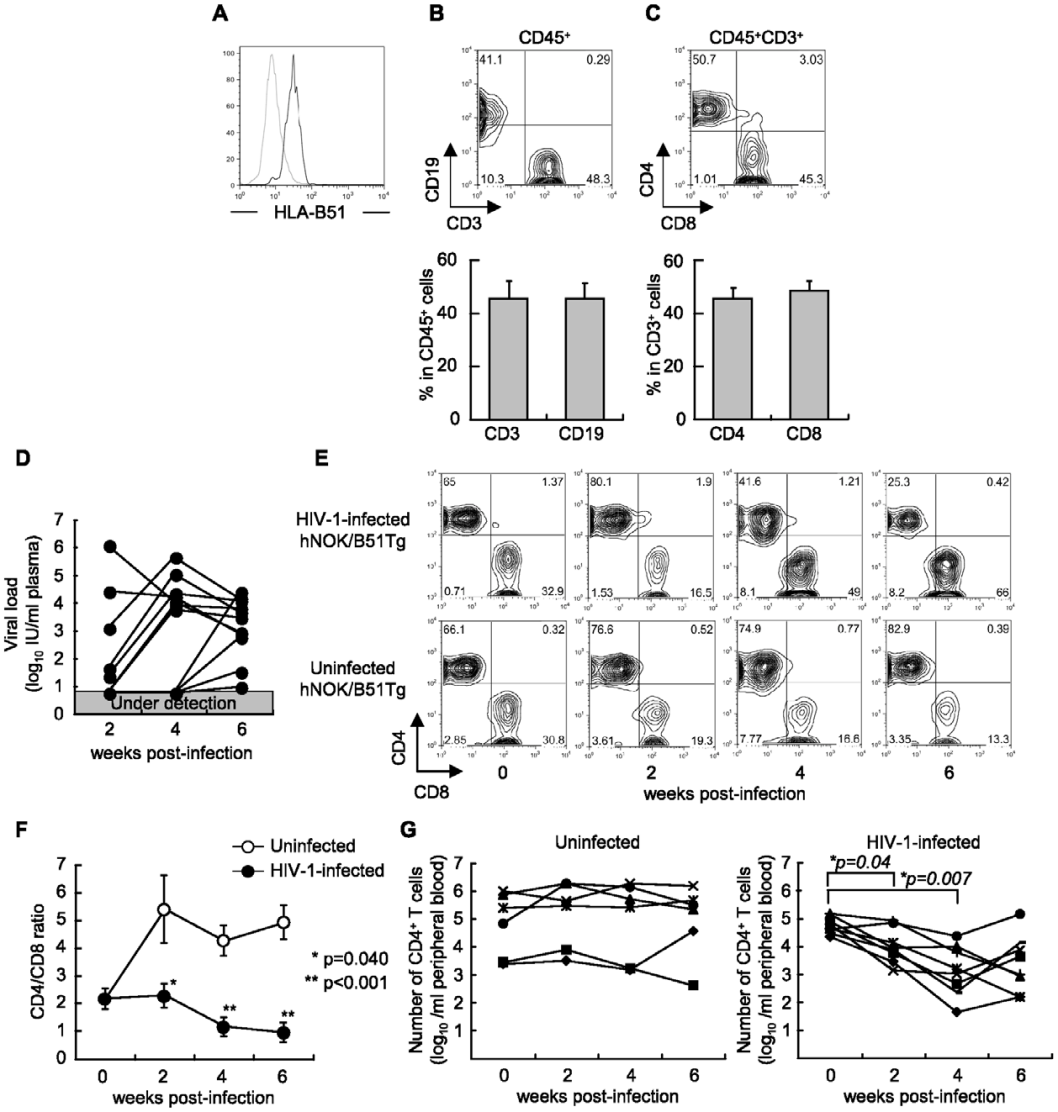
Establishment of a hNOK/B51Tg mouse model for the analysis of HIV-1 infections. NOK/B51Tg mice were established by backcrossing NOK mice with HLA-B*51:01 transgenic NOD/SCID mice, and then were analyzed for the expression of HLA-B51 molecules on their splenocytes. (A) Representative data on the expression of HLA-B51 molecules on splenocytes from a NOK/B51Tg mouse. Splenocytes were stained with TU109 mAB (black line) or isotype control (gray line). hNOK/B51Tg mice were analyzed for the frequency of reconstituted human cells at 10 weeks after the transplantation with human CD34^+^ cells. (B) Representative data and summary of the frequency of human CD19^+^ and CD3^+^ cells among the CD45^+^-gated subset in PBMCs (n = 18). (C) Representative data and summary of the frequency of human CD4^+^ and CD8^+^ T cells among CD45^+^/CD3^+^-gated subsets in PBMCs from hNOK/B51Tg mice (n = 18). hNOK/B51Tg mice were infected with HIV-1 at 14 weeks after the transplantation of CD34^+^ HSCs. (D) HIV-1-infected hNOK/B51Tg mice were analyzed for HIV-1 RNA and plasma viral load at 0, 2, 4, and 6 weeks post-infection (n = 11). (E) Representative data on human CD4^+^ and CD8^+^ T cell populations among CD45^+^/CD3^+^-gated subsets in PBMCs from an HIV-1-infected hNOK/B51Tg mouse (upper data) and from an uninfected one (lower data). (F) Summarized results on human CD4/CD8 T cell ratio at 0, 2, 4, and 6 weeks post-infection for PBMC from HIV-1-infected hNOK/B51Tg mice (n = 11, black circles) and from uninfected ones (n = 8, white circles). In uninfected hNOK/B51Tg mice, the proportion of human T cells in PBMC from the mice was observed from 14 weeks to 20 weeks after the transplantation. Asterisks indicate statistically significant differences (*p<0.05, HIV-1-infected hNOK/B51Tg mice *vs.* uninfected ones). Error bars represent SEMs. (G) The number of human CD4^+^ T cells in peripheral blood from HIV-1-infecetd hNOK/B51Tg (n = 8, right data) and uninfected ones (n = 6, left data) was determined. Asterisks indicate statistically significant differences (*p<0.05, HIV-1-infected hNOK/B51Tg mice at 2, 4, or 6 post-infection *vs.* hNOK/B51Tg mice before an HIV-1 infection).

We established NOK/B51Tg mice by backcrossing NOK mice with HLA-B*51:01 transgenic NOD/SCID mice. First, the expression of HLA-B51 molecules on their splenocytes (Figure 1A) and PBMCs (data not shown) was confirmed. Next, we generated hNOK/B51Tg and hNOK mice by transplanting human CD34^+^ HSCs derived from 17 cord blood samples into the liver of newborn NOK/B51Tg and NOK mice, and then analyzed them for the population of reconstituted human cells among the PBMCs of the mice at 10 weeks after the transplantation (Table 1). Human CD45^+^ cells were detected in both hNOK/B51Tg and hNOK mice, and the frequency of these cells was not significantly different between the 2 groups of mice (Figure S1A). Human CD3^+^ T cells and CD19^+^ B cells were detected among the human CD45^+^-gated subset of PBMCs from hNOK/B51Tg mice (Figure 1B). The human CD3^+^ T cell population included both CD4^+^ and CD8^+^ T cells (Figure 1C). The frequency of their cells was not significantly different between hNOK/B51Tg and hNOK mice (Figure S1B–E). The human T cells were maintained in the mice at least for 20 weeks after the transplantation, and transgenic expression of HLA-B51 did not influence the proportion of human T cells in hNOK/B51Tg mice compared with that in hNOK mice (Figure 1E, uninfected hNOK/B51Tg mouse, Figure S2A, uninfected hNOK mouse). These results indicate that human T cells were generated and maintained in hNOK/B51Tg and hNOK mice. On the other hand, human CD14^+^ cells including dendritic cells (DCs) and macrophages were hardly detected in either mouse strain (data not shown).

**Table 1:** Proportion of human immune cells in hNOK/B51Tg and hNOK mice.

				% nucleated cells				
Donor					hCD45 gated		hCD45/CD3 gated	
cord blood	No.	mouse	sex	hCD45+	CD3^+^	CD19^+^	CD4^+^	CD8^+^
1	1	NOK	F	1.68	88.1	7.05	59.4	40.3
	2	NOK	F	9.34	84.7	8.28	54.7	44.3
	3	NOK	M	5.13	98.6	0.36	61.6	37.6
	4	NOK	M	1.93	93.5	2.01	43.4	56.3
2	1	NOK/B51	F	5.69	22.6	70.3	36.0	58.6
	2	NOK/B51	F	2.46	61.6	34.5	31.6	66.4
	3	NOK/B51	F	7.92	10.2	83.0	20.6	74
	4	NOK/B51	F	7.66	21.3	74.6	20.3	69.3
	5	NOK/B51	M	4.2	27.8	63.6	33.9	55.2
3	1	NOK/B51	F	2.76	27.1	47.7	75.9	19.1
	2	NOK	M	1.06	32.6	46.1	71.9	19.0
	3	NOK/B51	M	14.8	74.9	19.2	49.0	42.0
4	1	NOK/B51	M	2.15	70.8	20.6	52.4	38.9
5	1	NOK/B51	F	3.3	72.2	16.1	34.7	51.4
6	1	NOK/B51	M	9.77	98.3	0.58	31.4	53.8
7	1	NOK/B51	F	8.76	6.87	81.5	67.1	31.5
	2	NOK	F	3.21	33.6	53.4	44.4	54.8
	3	NOK/B51	M	1.36	44.2	46.2	36.4	61.4
	4	NOK/B51	M	7.13	21.5	65.7	59.2	39.8
	5	NOK	M	3.72	61.7	28.7	47.2	52.2
8	1	NOK	M	19.5	66.7	25.3	59.2	35.8
9	1	NOK/B51	F	17.7	75.2	20.9	46.7	53.4
10	1	NOK	M	3.0	29.4	46.5	24.0	76.0
11	1	NOK/B51	M	10.6	48.3	39.5	50.7	45.3
12	1	NOK/B51	F	29.3	71.1	25.7	77.4	17.7
	2	NOK/B51	M	5.83	41.2	49.3	52.8	44.0
13	1	NOK/B51	M	6.77	28.4	58.1	46.0	54.0
	2	NOK	M	2.98	67.5	22.7	35.6	60.6
14	1	NOK	M	44.0	60.8	36.9	75.2	17.7
15	1	NOK	F	6.52	27.1	67.8	47.5	43.8
16	1	NOK/B51	F	4.03	19.5	73.2	37.6	61.7
	2	NOK	M	8.36	13.5	76.7	24.1	73.0
17	1	NOK	F	29.5	51.7	45.2	44.1	53.1
	2	NOK	F	29.6	62.1	33.9	49.3	49.3
	3	NOK	M	11.4	53.6	43.2	27.1	68.5

For generation of hNOK/B51Tg and hNOK mice, human CD34^+^ cells derived from 17 cord blood samples were transplanted into newborn NOK/B51Tg and NOK mice, respectively. PBMCs from hNOK/B51Tg (n = 19) and hNOK (n = 16) mice were analyzed for the proportion of human immune cells at 10 weeks after the transplantation of human CD34^+^ HSCs.

### Establishment of HIV-1-infected hNOK/B51Tg and hNOK mice

To establish an HIV-1-infected humanized mouse model, we infected hNOK/B51Tg and hNOK mice at 14 weeks after the transplantation of CD34^+^ HSCs with HIV-1_NL4-3_ and analyzed their plasma viral load every 2 weeks post-infection. Plasma viral RNA from hNOK/B51Tg mice was detected at 2 weeks post-infection and was maintained until 6 weeks post-infection (Figure 1D). Plasma viral RNA from hNOK mice was also detected; and there were no significant difference in the viral load from between hNOK/B51Tg and hNOK mice (data not shown). The proportion of human CD4^+^ and CD8^+^ T cells among the CD45^+^/CD3^+^-gated subsets of PBMCs from HIV-1-infected hNOK/B51Tg and hNOK mice was analyzed every 2 weeks post-infection by using flow cytometry and compared with that among PBMCs from uninfected hNOK/B51Tg and hNOK mice. Representative data from an HIV-1-infected hNOK/B51Tg mouse and an uninfected one is shown in Figure 1E. The proportion of human CD4^+^ T cells in the HIV-1-infected hNOK/B51Tg mouse gradually decreased from 2 weeks post-infection, whereas that in the uninfected hNOK/B51Tg mouse remained high. Summarized results of the CD4/CD8 ratio for the CD45^+^/CD3^+^-gated subsets among PBMCs from 11 HIV-1-infected and 8 uninfected hNOK/B51Tg mice showed that the CD4/CD8 ratio for HIV-1-infected hNOK/B51Tg mice was significantly decreased from 2 weeks post-infection as compared with that determined for the uninfected ones (Figure 1F). In addition, the number of human CD4^+^ T cells was decreased in HIV-1-infected hNOK/B51Tg mice but not in uninfected ones (Figure 1G). The proportion and number of human T cells among PBMC from HIV-1-infected hNOK mice was rather similar to those of these cells among PBMC from HIV-1-infected hNOK/B51Tg mice (Figure S2). These results showed that the progress of an HIV-1 infection in hNOK/B51Tg and hNOK mice was comparable to that found in patients with acute HIV-1 infection.

### Phenotypic analysis of human CD4^+^ T cells in HIV-1-infected and uninfected hNOK/B51Tg and hNOK mice

**Figure 2:**
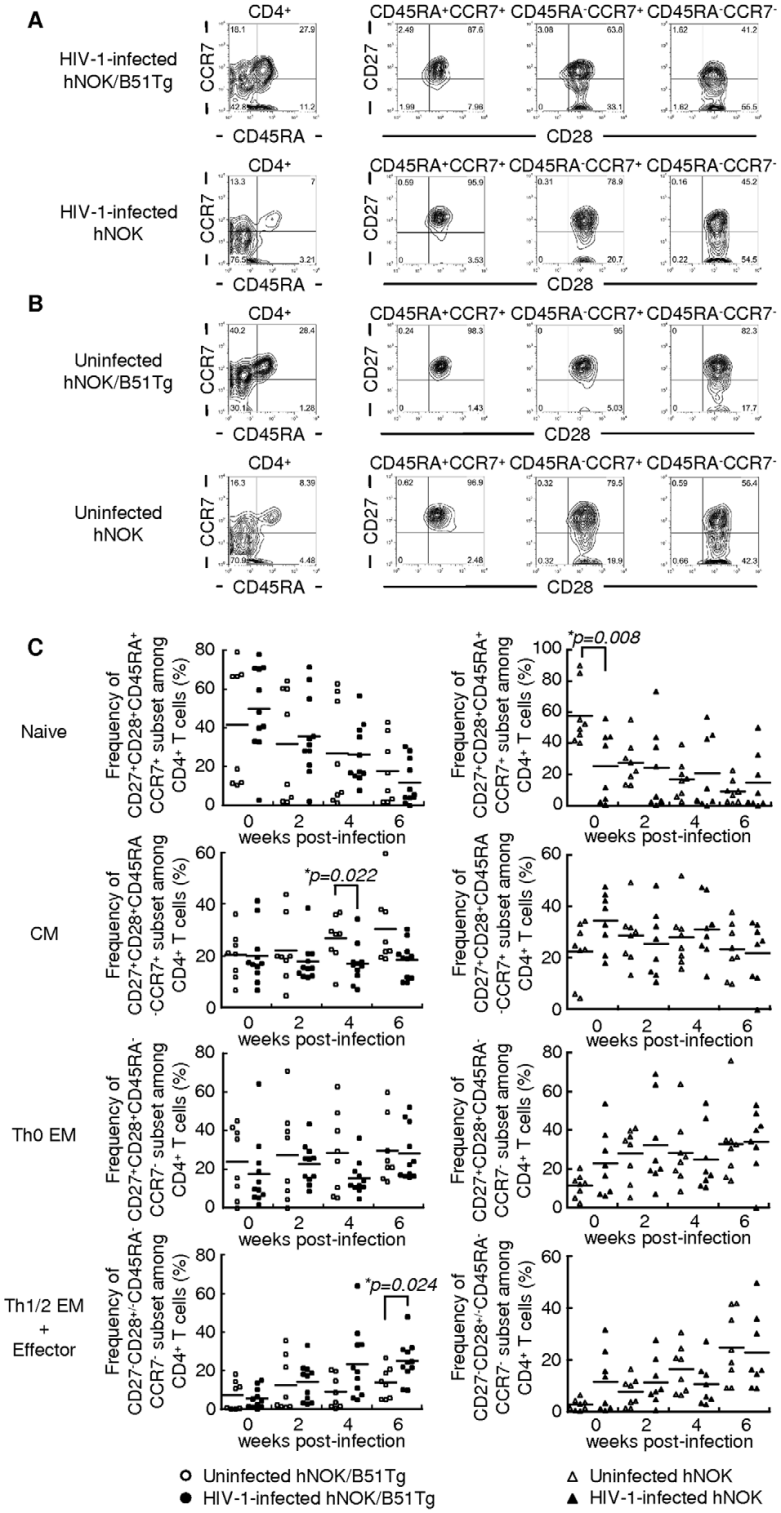
Phenotypic analysis of human CD4^+^ T cells in HIV-1-infected hNOK/B51Tg and hNOK mice as well as in uninfected ones. hNOK/B51Tg and hNOK mice were infected or not with HIV-1 at 14 weeks after the transplantation of CD34^+^ HSCs. Then human CD4^+^ T cells among PBMCs from HIV-1-infected and uninfected hNOK/B51Tg mice as well as HIV-1-infected and uninfected hNOK mice were analyzed for their expression of the following cell-surface markers: CD4, CD27, CD28, CD45RA, and CCR7. (A) Representative results of 5-color flow cytometric analysis of the human CD4^+^ T cell population in PBMCs from HIV-1-infected hNOK/B51Tg and HIV-1-infected hNOK mice at 6 weeks post-infection. (B) Same analysis as in “A” for uninfected hNOK/B51Tg and uninfected hNOK mice at 20 weeks after the transplantation of CD34^+^ HSCs. (C) Summarized results for the frequency of naive (CD27^+^CD28^+^CD45RA^+^CCR7^+^), central memory (CM:CD27^+^CD28^+^CD45RA^−^CCR7^+^), Th0 effector memory (Th0 EM:CD27^+^CD28^+^CD45RA^−^CCR7^−^), and Th1/2 effector memory (Th1/2 EM:CD27^−^CD28^+^CD45RA^−^CCR7^−^) plus effector (CD27^−^CD28^−^CD45RA^−^CCR7^−^) subsets among human CD4^+^ T cells in PBMCs from HIV-1-infected hNOK/B51Tg mice at 0, 2, 4, and 6 weeks post-infection (n = 11, black circles) and from uninfected ones at 14, 16, 18, and 20 weeks after the transplantation of CD34^+^ HSCs (n = 8, white circles) as well as those from HIV-1-infected hNOK mice (n = 8, black triangles) and from uninfected ones (n = 8, white triangles). Each symbol represents an individual mouse; and the mean value is shown as a horizontal solid line. Asterisks indicate statistically significant differences (*p<0.05, HIV-1-infected mice *vs.* uninfected ones).

Human peripheral CD4^+^ T cells are classified into the following 5 major populations based on their expression of 4 cell-surface marker: CD27^+^CD28^+^CD45RA^+^CCR7^+^ (naive subset), CD27^+^CD28^+^CD45RA^−^CCR7^+^ (central memory subset: CM), CD27^+^CD28^+^CD45RA^−^CCR7^−^ (Th0 effector memory subset: Th0 EM), CD27^−^CD28^+^CD45RA^−^CCR7^−^ (Th1/2 effector memory subset: Th1/2 EM), and CD27^−^CD28^−^CD45RA^−^CCR7^−^ (effector subset) [31]. To investigate the effect of an HIV-1 infection on the population of human CD4^+^ T cells in HIV-1-infected hNOK/B51Tg and hNOK mice, we analyzed the phenotype of these cells among PBMCs from the mice every 2 weeks post-infection. Representative data from HIV-1-infected hNOK/B51Tg and HIV-1-infected hNOK mice at 6 weeks post-infection (Figure 2A) and that from each uninfected ones at 20 weeks after the transplantation of CD34^+^ HSCs (Figure 2B), respectively, are shown; and summaries of the results from 11 HIV-1-infected and 8 uninfected hNOK/B51Tg mice as well as those from 8 HIV-1-infected and 8 uninfected hNOK mice are given in Figure 2C. The frequency of the CM subset among human CD4^+^ T cells from uninfected hNOK/B51Tg mice gradually increased every 2 weeks, whereas that for the HIV-1-infected hNOK/B51Tg mice was not altered during the HIV-1 infection. In addition, the frequency of Th1/2 EM and effector subsets among human CD4^+^ T cells for the infected mice gradually increased after the HIV-1 infection and was significantly higher at 6 weeks post-infection than that for the uninfected ones. These results show that the acute infection with the X4-tropic HIV-1 caused a depletion of the CM subset and a relative increase in the Th1/2 EM and effector subsets among human CD4^+^ T cells in the HIV-1-infected hNOK/B51Tg mice. On the other hand, there was no significant difference in the frequency of each subset among human CD4^+^ T cells between HIV-1-infected and uninfected hNOK mice (Figure 2C).

### Phenotypic analysis of human CD8^+^ T cells in HIV-1-infected and uninfected hNOK/B51Tg and hNOK mice

**Figure 3:**
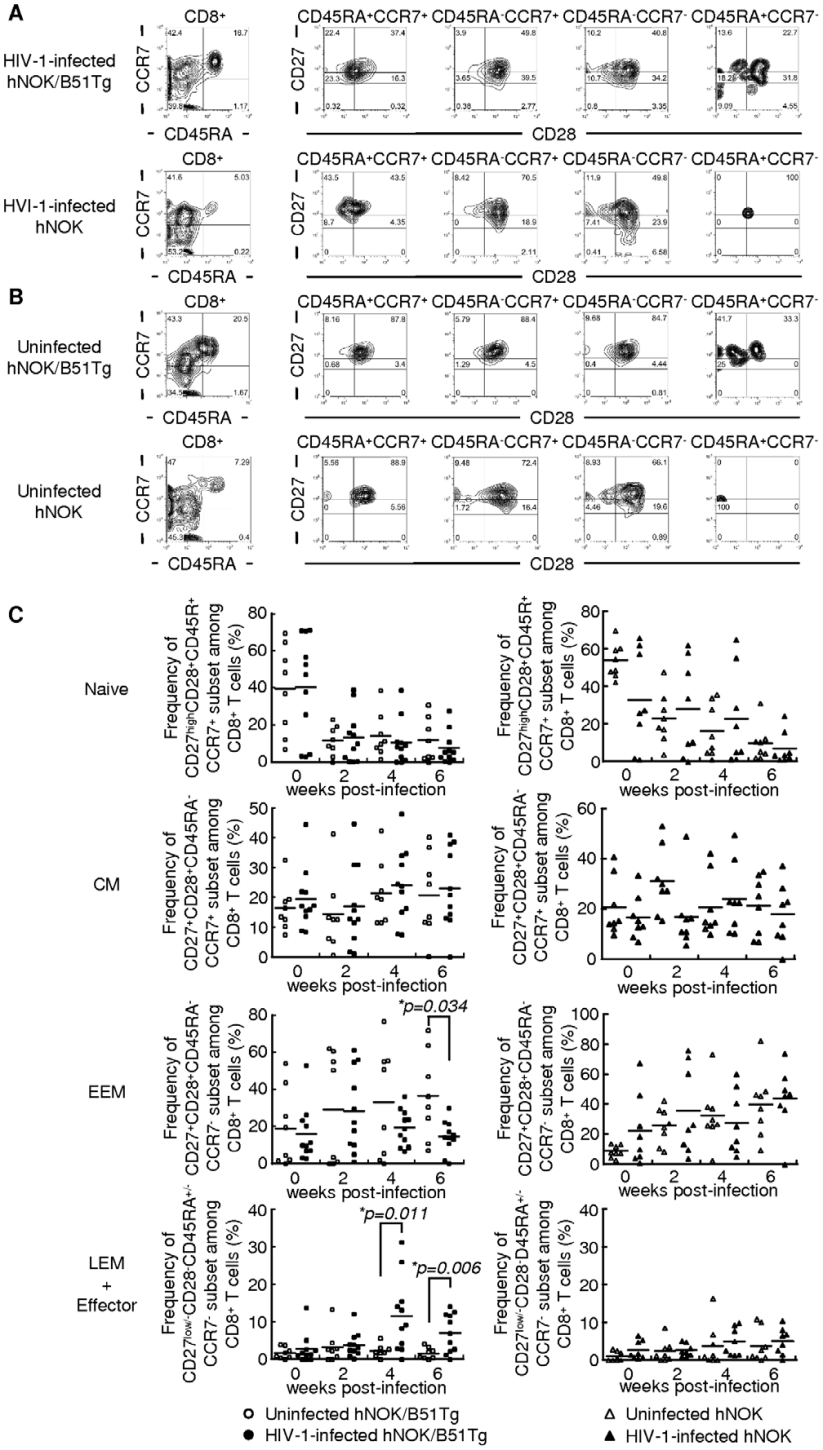
Phenotypic analysis of human CD8^+^ T cells in HIV-1-infected hNOK/B51Tg and hNOK mice as well as in uninfected ones. hNOK/B51Tg and hNOK mice were infected or not with HIV-1 at 14 weeks after the transplantation of CD34^+^ HSCs. Then human CD8^+^ T cells among PBMCs from HIV-1-infected and uninfected hNOK/B51Tg mice as well as HIV-1-infected and uninfected hNOK mice were analyzed for their expression of the following cell-surface markers: CD8, CD27, CD28, CD45RA, and CCR7. (A) Representative results of 5-color flow cytometric analysis of the human CD8^+^ T cell population in PBMCs from HIV-1-infected hNOK/B51Tg and HIV-1-infected hNOK mice at 6 weeks post-infection. (B) Same analysis as in “A” for uninfected hNOK/B51Tg and uninfected hNOK mice at 20 weeks after the transplantation of CD34^+^ HSCs. (C) Summarized results on the frequency of naive (CD27^high^CD28^+^CD45RA^+^CCR7^+^), central memory (CM: CD27^+^CD28^+^CD45RA^−^CCR7^+^), early effector memory (EEM: CD27^+^CD28^+^CD45RA^−^CCR7^−^), and late effector memory (LEM: CD27^low^CD28^−^CD45RA^+/−^CCR7^−^) plus effector (CD27^−^CD28^−^CD45RA^+/−^CCR7^−^) subsets of human CD8^+^ T cells among PBMCs from HIV-1-infected hNOK/B51Tg mice at 0, 2, 4, and 6 weeks post-infection (n = 11, black circles) and from uninfected ones at 14, 16, 18, and 20 weeks after the transplantation of CD34^+^ HSCs (n = 8, white circles) as well as those from HIV-1-infected hNOK mice (n = 8, black triangles) and from uninfected ones (n = 8, white triangles). Each symbol represents an individual mouse; and the mean value is shown as a horizontal solid line. Asterisks indicate statistically significant differences (*p<0.05, HIV-1-infected mice *vs.* uninfected ones).

Human peripheral CD8^+^ T cells are also classified into 5 major populations by the same 4 cell-surface markers, i.e., CD27^high^CD28^+^CD45RA^+^CCR7^+^ (naive subset), CD27^+^CD28^+^CD45RA^−^CCR7^+^ (central memory subset: CM), CD27^+^CD28^+^CD45RA^−^CCR7^−^ (early effector memory subset: EEM), CD27^low^CD28^−^CD45RA^+/−^CCR7^−^ (late effector memory subset: LEM), and CD27^−^CD28^−^CD45RA^+/−^CCR7^−^ (effector subset) [32], [33]. We previously showed that human CD8^+^ T cells can not differentiate into effector cells in hNOK mice stimulated with allo-antigen, because human CD8^+^ T cells are not educated by HLA in the mouse thymus [16]. To investigate the differentiation of human CD8^+^ T cells in hNOK/B51Tg and hNOK mice, we analyzed the phenotype of these cells among PBMCs from these mice. Representative data from uninfected hNOK/B51Tg and hNOK mice are shown in Figure 3B; and summaries of the results from 8 hNOK/B51Tg and 8 hNOK uninfected mice, in Figure 3C. Without stimulation there were no differences in the frequency of CD27^low^CD28^−^CD45RA^+/−^CCR7^−^ late effector memory and CD27^−^CD28^−^CD45RA^+/−^CCR7^−^ effector subsets among human CD8^+^ T cells between hNOK/B51Tg and hNOK mice. To clarify whether the differentiation of human CD8^+^ T cells was induced in hNOK/B51Tg and hNOK mice after an HIV-1 infection, we further analyzed the phenotype of human CD8^+^ T cells in PBMCs from the mice every 2 weeks post-infection. Representative results from HIV-1-infected hNOK/B51Tg and HIV-1-infected hNOK mice at 6 weeks post-infection are shown in Figure 3A; and summaries of the results from 11 HIV-1-infected hNOK/B51Tg and 8 HIV-1-infected hNOK mice, in Figure 3C. The frequency of LEM and effector subsets among human CD8^+^ T cells from HIV-1-infected hNOK/B51Tg mice at 4 and 6 weeks post-infection was significantly higher than that for the uninfected ones. On the other hand, a very low frequency of these subsets was detected in PBMCs from HIV-1-infected hNOK mice. These results suggest that human CD8^+^ T cells effectively differentiated into effector cells in the HIV-1-infected hNOK/B51Tg mice but not in the HIV-1-infected hNOK mice.

### Human effector CD8^+^ T cells expressing CXCR1 and/or CX3CR1 in HIV-1-infected hNOK/B51Tg mice

**Figure 4:**
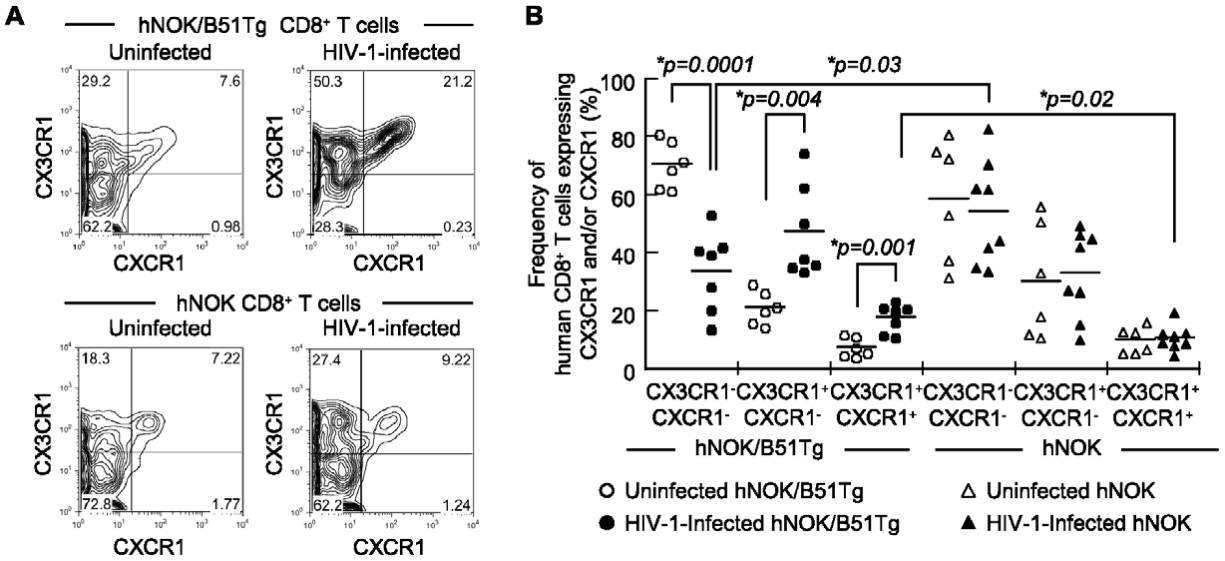
Expression of CX3CR1 and CXCR1 chemokine receptors on human CD8^+^ T cells from HIV-1-infected and uninfected hNOK/B51Tg mice and from HIV-1-infected and uninfected hNOK mice. Human CD8^+^ T cells from the spleens of HIV-1-infected hNOK/B51Tg and hNOK mice at 6 weeks post-infection as well as those from uninfected ones at 20 weeks after the transplantation of CD34^+^ HSCs were analyzed for the expression of CX3CR1 and CXCR1 molecules. (A) Representative results on CX3CR1 and CXCR1 co-expression on human CD8^+^ T cells from an HIV-1-infected hNOK/B51Tg mouse and from an uninfected one (upper data) as well as from an HIV-1-infected hNOK mouse and an uninfected one (lower data) are shown. (B) Summarized results on the frequency of human CD8^+^ T cells expressing CX3CR1 and/or CXCR1 from HIV-1-infected hNOK/B51Tg (n = 7, black circles) and uninfected (n = 6, white circles) mice as well as that for HIV-1-infected hNOK (n = 8, black triangles) and uninfected mice (n = 6, white triangles). Each symbol represents an individual mouse; and the mean value is shown as a horizontal solid line. Asterisks indicate statistically significant differences (*p<0.05).

Previous studies showed that CXCR1^+^ cells are predominantly found among human CD8^+^ T cells having effector function and that the expression of CXCR1 is positively correlated with that of perforin [34], [35]. An additional study revealed that CX3CR1 expression is closely associated with the development of effector functions in human CD8^+^ T cells and that CX3CR1-expressing CD8^+^ T cells represent a group of fully differentiated effector CD8^+^ T cells [36]–[38]. Based on the expression pattern of these molecules, we analyzed the frequency of human CD8^+^ T cells expressing CX3CR1 and/or CXCR1 from the spleens of HIV-1-infected and uninfected hNOK/B51Tg mice and of HIV-1-infected and uninfected hNOK mice at 6 weeks post-infection. Representative results from an HIV-1-infected hNOK/B51Tg mouse and an uninfected one as well as those from an HIV-1-infected hNOK mouse and an uninfected one are shown in Figure 4A. Only 3 subsets (CX3CR1^+^CXCR1^+^, CX3CR1^+^CXCR1^−^, and CX3CR1^−^CXCR1^−^) were detected in these mice. Summaries of the results from 7 HIV-1-infected and 6 uninfected hNOK/B51Tg mice as well as from 8 HIV-1-infected hNOK and 6 uninfected mice are shown in Figure 4B. The frequency of human CD8^+^ T cells expressing CX3CR1 and/or CXCR1 for HIV-1-infected hNOK/B51Tg mice was significantly higher than that for the uninfected ones, whereas the frequency of human CD8^+^ T cells expressing CX3CR1 and/or CXCR1 for the HIV-1-infected hNOK mice was not significantly higher than that for the uninfected ones. In addition, the frequency of human CD8^+^ T cells expressing both CX3CR1 and CXCR1 for the HIV-1-infected hNOK/B51Tg mice was significantly higher than that for the HIV-1-infected hNOK mice. These results support the idea that human CD8^+^ T cells having effector function were induced by the HIV-1 infection in hNOK/B51Tg mice.

## Discussion

We previously reported that human effector CD8^+^ T cells are not elicited in hNOK mice because, in this environment, human CD8^+^ T cells are educated by mouse MHC-peptide complexes in the thymus [16]. CD8 co-receptors bind to a nonpolymorphic region within the α3 domain of class I MHC proteins for the support of T cell recognition via TCR and suitable development of T cells in the thymus [39]–[41]. However, human CD8 hardly binds to the α3 domain of mouse class I MHC molecules [42], [43]. This fact indicates that human CD8^+^ T cells cannot be educated in the thymus of immunodeficient mice transplanted with only human CD34^+^ HSCs. Indeed, recent studies on NSG-HLA-A2/HHD mice infected with EBV show EBV-specific CD8^+^ T cell responses [13], [20]. We focused on elicitation of effector CD8^+^ T cells in hNOK/B51Tg mice after an HIV-1 infection in the present study.

We established hNOK/B51Tg mice by transplanting human CD34^+^ HSCs into HLA-B*51:01 transgenic NOK mice and investigated whether human effector CD8^+^ T cells could be elicited in these mice or in those infected with HIV-1. We found that human CD8^+^ T cells differentiated into effector cells in HIV-1-infected hNOK/B51Tg mice but not in HIV-1-infected hNOK mice, as evidenced by the induction of CD27^low^CD28^−^CD45RA^+/−^CCR7^−^ late effector memory and CD27^−^CD28^−^CD45RA^+/−^CCR7^−^ effector CD8^+^ T cells in the former mice but not in the latter. Thus, we demonstrated the differentiation of human CD8^+^ T cells into effector cells, although a previous study showed that only the CD45RA^−^CCR7^−^ CD8^+^ T cell subset is significantly increased in humanized NSG-HLA-A2/HHD mice after an infection with EBV [20]. These studies suggest that humanized mice established by transplanting human CD34^+^ cells into HLA-transgenic immunodeficient mice can produce mature CD8^+^ T cells that are capable of differentiating into effector T cells after viral antigen stimulation, because human CD8^+^ T cells can be educated by HLA molecules expressed in the thymus of these mice.

Since the effector function of virus-specific human CD8^+^ T cells is an important factor for the eradication of virus-infected cells, the elicitation of such virus-specific human CD8^+^ T cells in humanized mice is crucial for immunological studies on viral infection in such mice. Previous studies reported that IFN-γ production of EBV-specific T cells can be elicited in EBV-infected humanized NSG-HLA-A2/HHD mice [13], [20]. However, it remained unknown whether virus-specific T cell responses would be elicited in humanized mice after infection with HIV-1 targeting human CD4^+^ T cells. In the present study, we demonstrated that the frequency of CD27^low^CD28^−^CD45RA^+/−^CCR7^−^ late effector memory and CD27^−^CD28^−^CD45RA^+/−^CCR7^−^ effector CD8^+^ T cells was increased in HIV-1-infected hNOK/B51Tg mice. In addition, our data revealed that human CD8^+^ T cells expressing CX3CR1 and CXCR1 were induced in the HIV-1-infected hNOK/B51Tg mice. Human CD8^+^ T cells expressing the CD27^−^CD28^−^CD45RA^+/−^CCR7^−^ phenotype have effector function [44]. The expression of CX3CR1 and CXCR1 on human CD8^+^ T cells is associated with perforin expression in the cells and the effector function of them [34]–[38]. These findings strongly suggest that human CD8^+^ T cells with cytolytic effector function were elicited in hNOK/B51Tg mice after infection with HIV-1.

Antigen-specific T cell responses are found only occasionally in EBV- and HCV-infected humanized NSG mice [13], [15], whereas HIV-1-specific CD8^+^ T cells from HIV-1-infected NOG and NSG mice are detected only when spleen cells are stimulated with HIV-1 gag and env peptides *in vitro* [26], [30]. Since human T cells should be educated by HLA molecules expressed in the thymus of the mice for elicitation of their suitable function, there are 2 possibilities to explain the elicitation of human effector responses in the mice. Marodon et al. reported that V-J combination distribution of human T cells is already present before TCR-MHC selection in the thymus of NSG mice [15]. In addition, Pham et al. reported a similar distribution of human T cell receptor β chain families in the thymus of NSG mice and in human thymus, suggesting the selection of functional human T cells reconstituted in these mice [45]. In addition, some thymic selection of human T cells occurs on mouse MHC, as evidenced by a slightly increased weak proliferation of human T cells toward mismatched mouse dendritic cells as compared with that toward host ones [8]. Besides we found that human T cells were maintained for 20 weeks at least in hNOK mice. Considering that CD8^+^ T cells have a drastically shorter life span in the absence of peripheral MHC class I [46], human CD8^+^ T cells can only interact with peripheral mouse MHC for their survival in the periphery of hNOK mice; whereas human CD8^+^ T cells cannot differentiate into effector cells in these mice because of the education by mouse MHC [42], [43]. Another possibility is that functional human CD8^+^ T cells are elicited in NSG and NOK mice if those cells are educated by donor-derived DCs in the thymus of the mice, as suggested in previous studies [13], [15]. However, considering that human effector CD8^+^ T cells were hardly elicited in HIV-1-infected hNOK mice, we assume that human CD8^+^ T cells in the hNOK/B51Tg mice were educated by HLA-B51 molecules in the thymus of the mice and differentiated into effector cells in response to stimulation by HIV-1 antigen; although it remains possible that a few T cells were educated by mouse MHC and donor-derived DCs in the thymus of the mice.

Since the frequency of HLA-B51 allele in the case of Japanese population is about 20% [47], we could not have enough HLA-B51-positive donor HSCs for the transplantation of CD34^+^ HSCs into NOK/B51Tg mice. A previous study demonstrated that double cord blood transplantation results in increased engraftment levels in the bone marrow and peripheral blood in comparison with recipients given a single unit, implying that the increased level of engraftment might be the result of an undefined graft-*vs.*-graft stimulatory effect [48]. However, the frequency of reconstituted human CD45^+^ cells was not significantly different between hNOK/B51Tg and hNOK mice, suggesting no activation of HSCs by alloantigen in hNOK/B51Tg mice. In addition, there was no *de novo* acute graft-versus-host (GVH) alloreaction in the hNOK/B51Tg mice at 20 weeks at least after the transplantation of the CD34^+^ HSCs. Considering that the elicitation of effector CD8^+^ T cells was demonstrated in HIV-1-infected hNOK/B51Tg mice, these results support the idea that cross-reactive human CD8^+^ T cell responses against viral antigen were elicited in hNOK/B51Tg mice infected with HIV-1. However, there are some arguments about an allo-HLA reactivity of virus-specific T cells. Amir et al. demonstrated that a substantial proportion of virus-specific T cells exert allo-HLA reactivity *in vitro* [49]. On the other hand, Melenhorst et al. reported that allogeneic virus-specific T cells with HLA alloreactivity do not produce GVHD in humans despite of *in vitro* recognition of HLA-mismatched targets [50]. These studies suggest that virus-specific T cells have different features for HLA alloreactivity between *in vivo* and *in vitro* circumstances. In the case of hNOK/B51Tg mice transplanted with HLA-B51-negative CD34^+^ HSCs, we consider that precursor human CD8^+^ T cells are educated by HLA-B51 molecule in the thymus of hNOK/B51Tg mice and then functional CD8^+^ T cells having the ability to differentiate into effector cells are selected in them. On the other hand, functional human CD8^+^ T cells are hardly selected in hNOK mice, since precursor human CD8^+^ T cells are educated by mouse MHC molecules in the thymus of these mice. In fact, a very low frequency of human effector CD8^+^ T cells were detected in hNOK mice after an infection of HIV-1. We speculate that precursor human CD8^+^ T cells derived from HLA-mismatched CD34^+^ HSCs interact with HLA-B51 much better than with mouse MHC in the thymus, because human CD8 can bind to HLA but not to mouse MHC.

In the present study, we could not analyze HIV-1-specific CD8^+^ T cell responses in hNOK/B51Tg mice because of the low frequency of human T cell reconstitution in the mice. Since the expression of stem cell factor (SCF), which facilitates HSC proliferation and survival, increases in mice with irradiation [51], human CD34^+^ HSCs are generally transplanted into irradiated-immunodeficient mice. However, sub-lethal irradiation of newborn mice leads to deficient weight gain and might cause damage to neurons in the developing brain [52]. We established hNOK/B51Tg mice by transplanting hCD34^+^ HSCs into non-irradiated hNOK/B51Tg mice to exclude those types of damage to the mice. Therefore, the frequency of human T cell reconstitution in the hNOK/B51Tg mice was lower than that for humanized NSG mice [6]. Recent studies showed that the frequency of human cell reconstitution in human transgenic membrane-bound stem cell factor (SCF) NSG mice (NSG-Tg (hu-mSCF)) is higher than that in irradiated NSG mice [53] and that SCF administration improves the recovery of thymopoiesis following HSC transplantation in Rag2^−/−^γC^−/−^ mice [54]. These findings suggest that SCF administration to hNOK/B51Tg mice may enhance human HSC engraftment and T cell reconstitution in these mice.

It is well known that HIV-1-infected memory CD4^+^ T cells are severely depleted in number during the acute phase of an HIV-1 infection [55]. Indeed, our mouse model confirmed this acute loss of CD4^+^ T cells. Therefore, it is likely that human CD8^+^ T cells are not effectively primed by HIV-1 in hNOK/B51Tg mice because of the loss of support by helper CD4^+^ T cells. Previous studies revealed that the Nef-mediated down-regulation of HLA class I molecules impairs the cytolytic activity of HIV-1-specific CTLs toward HIV-1-infected CD4^+^ T cells [56], [57]. On the other hand, HIV-1-specific CTLs can effectively kill Nef-defective HIV-1 mutant-infected CD4^+^ T cells and completely suppress the replication of the HIV-1 mutant [57], [58]. These studies suggest that the HIV-1-specific CTL responses may be effectively induced in hNOK/B51Tg mice by using this Nef-defective HIV-1.

In an acute HIV-1 infection, HIV-specific effector CD8^+^ T cells are highly elicited from naive T cells [59]. In addition, the frequency of human CD8^+^ T cells expressing CX3CR1 and perforin is dramatically increased for HIV-1-positive individuals compared with that for uninfected ones [60]. The present study showed that effector CD8^+^ T cell subsets and CD8^+^ T cells expressing CX3CR1/CXCR1 were induced in hNOK/B51Tg mice after an infection with HIV-1, indicating that the T cell responses to an HIV-1 infection in hNOK/B51Tg mice are similar to those found in patients with an acute HIV-1 infection. In addition, CXCR4-tropic (X4) virus HIV-1_NL4-3_ strongly depleted the central memory CD4^+^ T cell subset in the hNOK/B51Tg mice after an infection with HIV-1. A similar finding was reported based on a previous X4 HIV-1-infected humanized mouse study that showed quick depletion of CD4^+^ thymocytes and of both CD45RA^+^ naive and CD45RA^−^ memory CD4^+^ T cells [61]. These studies confirmed the findings of loss of memory CD4^+^ T cell in HIV-1-infected individuals. On the other hand, the viral load was not decreased much during 6 weeks post-infection in HIV-1-infected hNOK/B51Tg mice; and there was no significant difference in the viral road between HIV-1-infected hNOK/B51Tg and HIV-1-infected hNOK mice. HLA-mismatched CD34^+^ HSCs were transplanted into hNOK/B51Tg mice in the present study, implying that the function of human CD8^+^ T cells in the mice might be weaker than that in human individuals. In addition, the number of human CD8^+^ T cells gradually decreased in both hNOK/B51Tg and hNOK mice in spite of the stable number of human CD4^+^ T cells. Human CD8^+^ T cells require 2 main cytokines, IL-15 for their proliferation and IL-7 for their survival [62], [63]. However, IL-15 is species-specific [64], meaning that mouse IL-15 would have no effect on human CD8^+^ T cells in humanized mice. Thus, human CD8^+^ T cells can not eliminate the virus since the CD8^+^ T cells could not proliferate in hNOK/B51Tg mice after an infection of HIV-1. These mouse models would be a useful tool for *in vivo* study of human immune responses to HIV-1 and virus pathogenicity, if the problems associated with reconstituted human T cells in humanized mice are resolved.

In summary, we established HLA-B*51:01 transgenic humanized mice and demonstrated the elicitation of human effector CD8^+^ T cells, but no GVH reaction, in the mice after infection with HIV-1. The human CD8^+^ T cell responses in the mice reflected the progress of an HIV-1 infection comparable to that found in patients with an acute HIV-1 infection. hNOK/B51Tg mice represent a new animal model for the study of HIV-1 infection, and transplantation with HLA-matched HSCs and improved reconstitution with human T cells in hNOK/B51Tg mice will facilitate future studies on HIV-1-specific CTL responses in such mice after an infection with HIV-1.

## Materials and Methods

### Establishment of HLA-B*51:01 transgenic (Tg) humanized mice

NOK/B51Tg mice were established by backcrossing NOK mice [16] with NOD/SCID/B51Tg mice. The NOD/SCID/B51Tg mice were established by backcrossing NOD/SCID mice with HLA-B*51:01 transgenic C3H/HeJ mice [65] for 10 generations. These mice were maintained under specific pathogen-free conditions in the Center for Animal Resources and Development at Kumamoto University. Animal experiments were approved by the Ethics Committee for Animal Care and Use of Kumamoto University (Approval ID, B23-181 R1: Analysis of immune responses against viral diseases by using humanized and HLA transgenic mice). Human cord blood (hCB) was purchased from Riken Cell Bank (Tsukuba, Japan). Human CD34^+^ HSCs were purified from hCB by using a Direct CD34 Progenitor Cell Isolation Kit and an LS Column (Miltenyi Biotec, Gladbach, Germany). The average purity of human CD34^+^ cells was approximately 90%. hNOK and hNOK/B51Tg mice were generated by injecting purified human CD34^+^ HSCs (5×10^4^∼1×10^5^ cells/mouse) into the liver of newborn NOK and NOK/B51Tg mice, respectively. We used 17 different cord blood samples to generate humanized mice. All hCB cells transplanted into the mice were HLA-B51 negative.

### Infection with and titration of HIV-1

A virus solution of HIV-1_NL4-3_ was prepared and titrated as previously described [66]. The virus solution containing 2000 blue cell-forming units (BFU) was injected intraperitoneally into 14- to 16-week-old hNOK/B51Tg or hNOK mice. Viral RNA was extracted from 50 µl plasma of HIV-1-infected hNOK/B51Tg mice by using a QIAamp MinElute virus spin kit (QIAGEN, Tokyo, Japan). The RNA was reverse transcribed and amplified by using a SuperScript™ III Platinum SYBR Green One-Step qRT-PCR Kit (invitrogen, Carlsbad, CA) with HIV-1_NL4-3_ consensus primers (forward, 5′-CCCATCAAAAGACTTAATAGCAGAAATACA-3′; and reverse, 5′-TTCTTGCATATTTTCCTGTTTTCA-3′). The amplified PCR products were quantitatively monitored by using an Applied Biosystems ABI Prism 7500. HIV TYPE I NATtrol™ (viroquest, Osaka, Japan) was used as control RNA for quantification.

### Flow cytometric analysis

Peripheral blood mononuclear cells (PBMCs) were taken from a tail vein of HIV-1-infected or uninfected hNOK/B51Tg mice as well as from HIV-1infected or uninfected hNOK mice. Splenocytes were harvested from the spleens of these mice at 6 weeks post-infection. The PBMCs or splenocytes were stained with various combinations of monoclonal antibodies (mAbs) such as PECy7-labeled anti-CD45, APC-labeled anti-CD4, APC-Cy7-labeled anti-CD4, AmCyan-labeled anti-CD8, Pacific blue-labeled anti-CD8, PE-labeled anti-CD19, PE-Cy7-labeled anti-CCR7, FITC-labeled anti-CD45RA, APC-eFluor®780-labeled anti-CD27, PE-labeled anti-CD28 mAbs (BD Biosciences, San Diego, CA), ECD-labeled anti-CD3 mAb (Beckman Coulter, Fullerton, CA), APC-labeled anti-CXCR1, and PE-Cy7-labeled anti-CX3CR1 mAb (Biolegend, San Diego, CA). To analyze the phenotype of human T cells reconstituted in hNOK/B51Tg and hNOK mice, we first stained human T cells with anti-CCR7 mAb for 30 min at room temperature, and subsequently with specific antibody against surface markers for 30 min at 4°C. The cells were washed twice with PBS containing 10% fetal calf serum (FCS; Sigma-Aldrich, St. Louis, MO). The stained cells were analyzed by using a FACSCant II flow cytometer (BD Biosciences, San Jose, CA). All flow cytometric data were analyzed by using FlowJo software (Tree Star, Inc, Ashland, OR).

### Statistical analysis

Results shown as bar graphs were expressed as the means ± S.E.M. Comparisons of 2 groups were performed with Student's *t* test. In all analyses, a two-tailed probability of less than 5% (i.e. *P<0.05) was considered statistically significant.

## Supporting Information

Figure S1 **Proportion of human reconstituted cells in PBMC from hNOK/B51Tg and hNOK mice at 10 weeks after CD34^+^ cell transplantation.** hNOK/B51Tg and hNOK mice were established by transplanting human CD34^+^ HSCs into NOK/B51Tg and NOK mice, respectively. The proportion of (A) human CD45^+^, (B) CD45^+^-gated CD3^+^, (C) CD45^+^-gated CD19^+^, (D) CD45^+^/CD3^+^-gated CD4^+^, and (E) CD45^+^/CD3^+^-gated CD8^+^ cells were analyzed at 10 weeks after the transplantation of CD34^+^ HSCs by using FACS. Each symbol represents a hNOK/B51Tg mouse (n = 19, black circles) and a hNOK mouse (n = 16, black triangles); and the mean value is shown as a horizontal solid line. There was no significance of the proportion of each reconstituted human cells between the 2 groups of mice.
(TIF)

Figure S2 **Establishment of a hNOK mouse model for the analysis of HIV-1 infections.** hNOK mice were infected with HIV-1 at 14 weeks after the transplantation of human CD34^+^ HSCs. (A) Representative data on human CD4^+^ and CD8^+^ T cell populations among CD45^+^/CD3^+^-gated subsets in PBMCs from an HIV-1-infected hNOK mouse at 0, 2, 4, and 6 weeks post-infection (upper data) and from an uninfected one at 14, 16, 18, and 20 weeks after the transplantation of CD34^+^ HSCs (lower data). (B) Summarized results on human CD4/CD8 T cell ratio at 0, 2, 4, and 6 weeks post-infection for PBMC from HIV-1-infected hNOK mice (n = 8, black triangles) and from uninfected ones (n = 8, white triangles). In uninfected hNOK mice, the proportion of human T cells in PBMC from the mice was observed from 14 weeks to 20 weeks after the transplantation. Asterisks indicate statistically significant differences (*p<0.05, HIV-1-infected hNOK mice *vs.* uninfected ones). Error bars represent SEMs. (C) The number of human CD4^+^ T cells in peripheral blood from HIV-1-infecetd hNOK (n = 8, right data) and uninfected ones (n = 4, left data). Asterisks indicate statistically significant differences (*p<0.05, HIV-1-infected hNOK mice at 2, 4, or 6 post-infection *vs.* hNOK mice before an HIV-1 infection).
(TIF)
