# Supplementary figures and images for: Effective Elicitation of Human Effector CD8+ T Cells in HLA-B*51:01 Transgenic Humanized Mice after Infection with HIV-1
Source: PLoS One. 2012 Aug 6;7(8):e42776. doi: 10.1371/journal.pone.0042776 (PMC3412802; doi:10.1371/journal.pone.0042776)

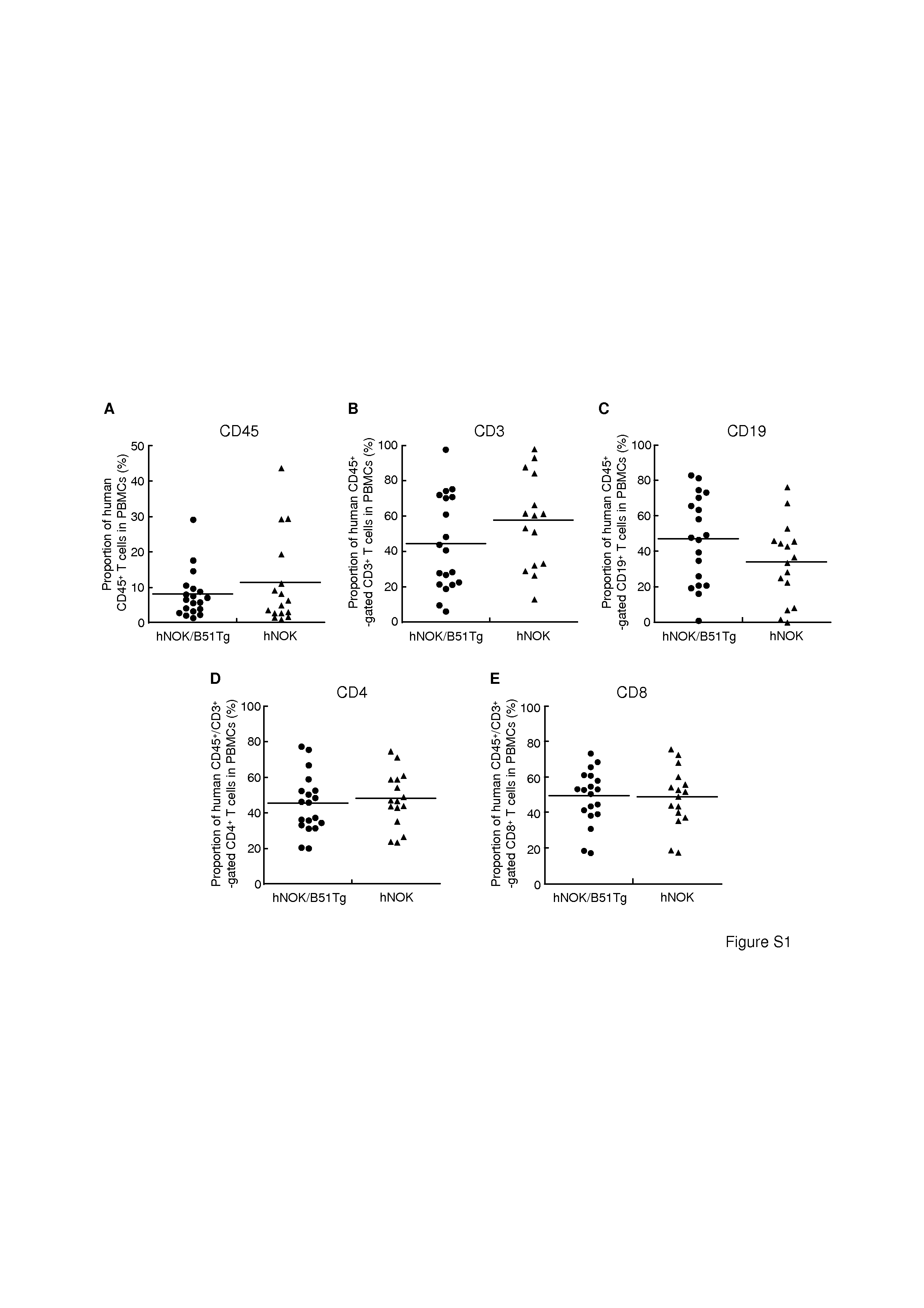

Supplement: Figure S1 — Proportion of human reconstituted cells in PBMC from hNOK/B51Tg and hNOK mice at 10 weeks after CD34+ cell transplantation. hNOK/B51Tg and hNOK mice were established by transplanting human CD34+ HSCs into NOK/B51Tg and NOK mice, respectively. The proportion of (A) human CD45+, (B) CD45+-gated CD3+, (C) CD45+-gated CD19+, (D) CD45+/CD3+-gated CD4+, and (E) CD45+/CD3+-gated CD8+ cells were analyzed at 10 weeks after the transplantation of CD34+ HSCs by using FACS. Each symbol represents a hNOK/B51Tg mouse (n = 19, black circles) and a hNOK mouse (n = 16, black triangles); and the mean value is shown as a horizontal solid line. There was no significance of the proportion of each reconstituted human cells between the 2 groups of mice. (TIF) [file pone.0042776.s001.tif]

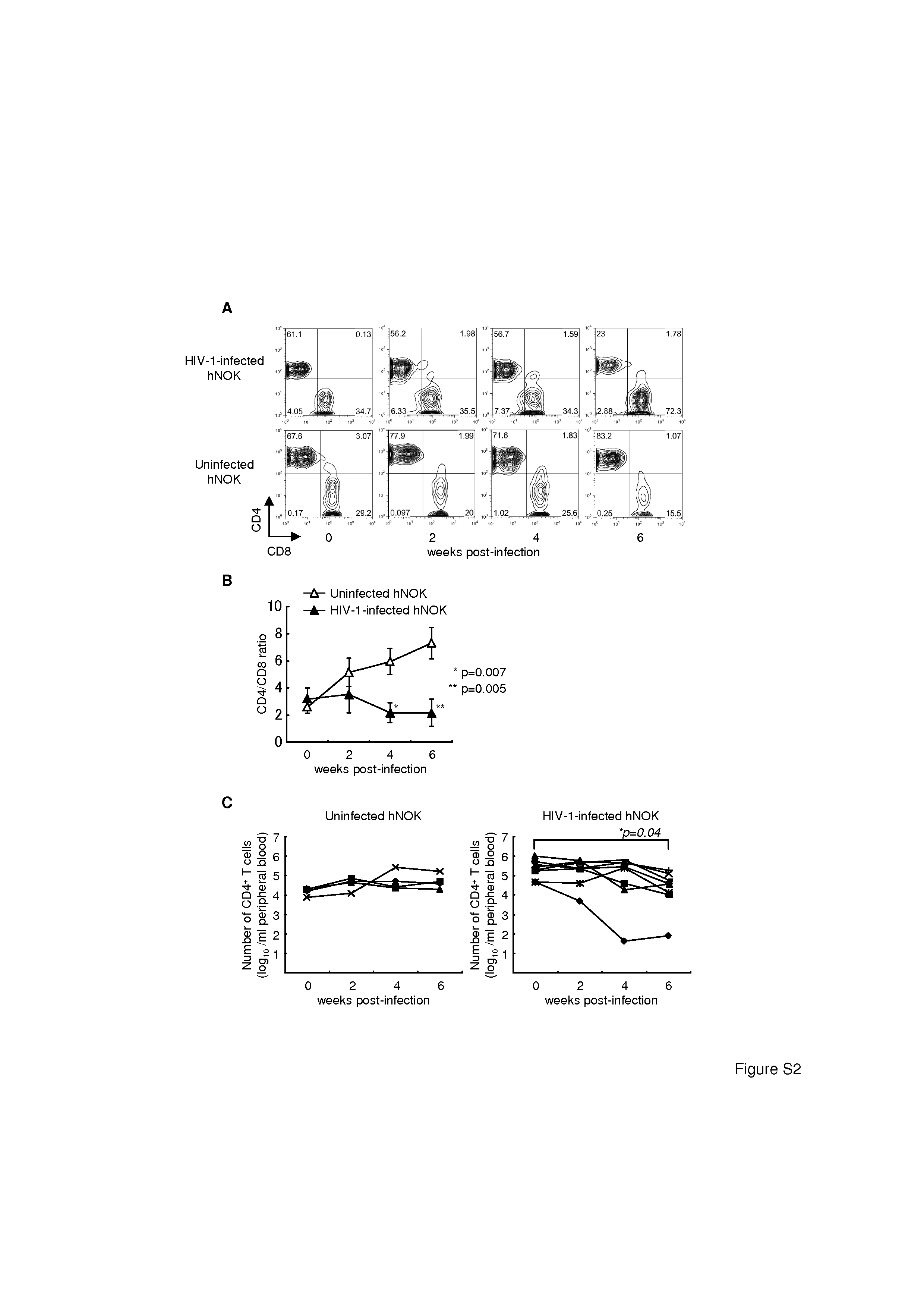

Supplement: Figure S2 — Establishment of a hNOK mouse model for the analysis of HIV-1 infections. hNOK mice were infected with HIV-1 at 14 weeks after the transplantation of human CD34+ HSCs. (A) Representative data on human CD4+ and CD8+ T cell populations among CD45+/CD3+-gated subsets in PBMCs from an HIV-1-infected hNOK mouse at 0, 2, 4, and 6 weeks post-infection (upper data) and from an uninfected one at 14, 16, 18, and 20 weeks after the transplantation of CD34+ HSCs (lower data). (B) Summarized results on human CD4/CD8 T cell ratio at 0, 2, 4, and 6 weeks post-infection for PBMC from HIV-1-infected hNOK mice (n = 8, black triangles) and from uninfected ones (n = 8, white triangles). In uninfected hNOK mice, the proportion of human T cells in PBMC from the mice was observed from 14 weeks to 20 weeks after the transplantation. Asterisks indicate statistically significant differences (*p<0.05, HIV-1-infected hNOK mice vs. uninfected ones). Error bars represent SEMs. (C) The number of human CD4+ T cells in peripheral blood from HIV-1-infecetd hNOK (n = 8, right data) and uninfected ones (n = 4, left data). Asterisks indicate statistically significant differences (*p<0.05, HIV-1-infected hNOK mice at 2, 4, or 6 post-infection vs. hNOK mice before an HIV-1 infection). (TIF) [file pone.0042776.s002.tif]
